# Design of a broadband circularly polarised uniplanar crossed-dipole antenna

**DOI:** 10.1038/s41598-024-56947-w

**Published:** 2024-04-03

**Authors:** Heesu Wang, Yong Bae Park, Ikmo Park

**Affiliations:** 1 Department of Electrical and Computer Engineering, Ajou University, Suwon, 16499 Republic of Korea; 2 Department of AI Convergence Network, Ajou University, Suwon, 16499 Republic of Korea

**Keywords:** Engineering, Electrical and electronic engineering

## Abstract

This study presents the design of a uniplanar crossed-dipole antenna with broadband characteristics. The antenna comprises a pair of identical crossed-dipole arms printed on the same plane of a dielectric substrate. The crossed-dipole arms are corner-cut fat dipoles that are perpendicular to each other and connected with a bent stripline to generate circularly polarised (CP) radiations. A wide dipole arm was used to improve impedance matching and widen the axial ratio (AR) bandwidth. Additionally, the corner of each dipole arm was cut into a triangular shape to broaden the impedance and AR bandwidths further. The antenna in free space is excited via a wideband microstrip-to-parallel stripline tapered balun to reduce the effect of leakage current on the coaxial cable. Experiments and full-wave electromagnetic simulations were employed to design, verify, and validate the antenna design. The antenna, having an overall size of 45 × 45 × 0.508 mm^3^ (0.4 × 0.4 × 0.0045 λ_L_^3^ where λ_L_ is the lowest frequency in the 3-dB AR bandwidth), demonstrates the following measured performances: an |S_11_|< − 10 dB impedance bandwidth of 2.53–9.14 GHz (113.3%), a 3 dB AR bandwidth of 2.65–7.75 GHz (98.1%), and peak gain of 3.7 dBic at 6.6 GHz.

## Introduction

The tremendous interest in circularly polarised (CP) antennas as compared with linearly polarised (LP) antennas in recent times is because CP antennas possess advantages such as enhanced robustness to multipath distortion and polarisation mismatch losses. Due to these superior qualities, CP antennas are continually in demand for many wireless communication applications^1–4^. The rudimentary principle in generating CP entails producing two orthogonal electric fields with equal amplitude and a 90° phase difference^5–7^. In some designs, two or more orthogonal dipoles of unequal length are used to achieve a 90° phase difference^8,9^. For example, a cavity-backed multi-dipole antenna consisting of two longer dipoles and two shorter dipoles produced an axial ratio (AR) bandwidth of 7.55%^8^. Furthermore, in Ref.^9^, a 5.6% AR bandwidth was achieved when two pairs of parallel folded dipoles were orthogonally placed in a square contour and excited to the same amplitude with a 90° phase difference. Alternatively, a 90° phase delay line in the form of a vacant-quarter ring printed on both sides of a substrate was customarily integrated between two orthogonal dipoles to form single-feed crossed-dipole antennas^10–14^. Traditional crossed-dipole antennas composed of thin dipoles offer limited bandwidth; for example, the printed crossed-dipole antenna in Ref.^14^ generated an AR bandwidth of 15.6%. Accordingly, the increasing demands of the bandwidth of modern wireless communication systems and the progress in antenna design have prompted the development of wideband crossed-dipole antennas.

The research and development of crossed-dipole antennas have proliferated due to the merits of a simple structure, wide bandwidth, flexible shape, and superior CP characteristics^10–28^. Recently, several techniques have been proposed to widen the AR bandwidth of crossed-dipole antennas. A common approach for achieving wide CP bandwidth with a crossed-dipole antenna is the use of wide planar dipoles instead of thin linear dipoles. For example, a wideband CP crossed dipole consisting of rectangular dipoles produced a 27% AR bandwidth^16^. Crossed-dipole antennas made of wide planar radiators, such as stepped dipoles^18^ and bowties^27^, have generated wide CP bandwidths of 55.1% and 51%, respectively. Some approaches incorporate additional radiators, such as parasitic elements around the dipole, to increase the AR bandwidth. A crossed-dipole antenna with a single asymmetric loop demonstrated an AR bandwidth of 53.4%^11^, whereas the crossed-dipole antenna with four loop resonators in Ref.^28^ produced an AR bandwidth of 28.6%. Furthermore, a combination of the aforementioned techniques, which use wide planar crossed dipoles and parasitic elements, is very much prevalent in AR bandwidth enhancement. A single parasitic bowtie element was incorporated with a bowtie-shaped crossed dipole to achieve an AR bandwidth of 58.6%^22^. Accordingly, the previously mentioned approaches successfully widen the AR bandwidth of crossed dipoles at the expense of employing additional, larger radiators that increase the size and fabrication cost. In addition, the antennas have a complex structure that may increase the difficulty of large-scale production.

Therefore, this study presents a uniplanar crossed-dipole antenna with broadband characteristics. The antenna comprises a pair of identical crossed-dipole arms that are printed uniquely on the same plane of a dielectric substrate, thereby minimising the antenna design and fabrication complexity^29^. The antenna employs two pairs of corner-cut fat-dipole arms to improve impedance matching and generate a wide AR bandwidth. The antenna was optimised with ANSYS High-Frequency Simulation Software (HFSS), and the measurements were verified to demonstrate the following performance characteristics. The antenna, having an overall size of 45 × 45 × 0.508 mm (0.4 × 0.4 × 0.0045 λ_L_, where λ_L_ is the lowest frequency in the 3-dB AR bandwidth), demonstrated an |S_11_|< − 10 dB bandwidth of 2.53–9.14 GHz (113.3%), a 3-dB AR bandwidth of 2.65–7.75 GHz (98.1%), and a peak gain of 3.7 dBic at 6.6 GHz.

### Antenna geometry and design

**Figure 1:**
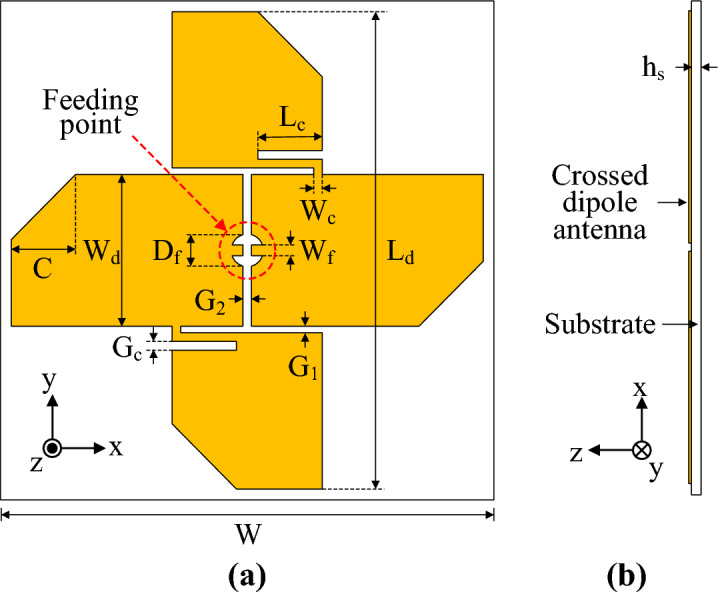
Geometry of a crossed-dipole antenna: (**a**) top view and (**b**) side view.

The geometry of the uniplanar crossed-dipole antenna consists of two orthogonal fat-dipole pairs and a dielectric substrate, as shown in Fig. 1. The crossed dipoles are composed of two dipole arms that are orthogonal to each other and are connected with a bent stripline for CP production. The dielectric substrate material is Rogers RO4003, which has the properties *ε*_*r*_ = 3.38 and *tanδ* = 0.0027. Fat dipoles are used for crossed-dipole arms to improve the AR and impedance matching. The corners of each fat-dipole arm were cut in a triangular shape to expand the AR bandwidth, and the crossed-dipole antenna was optimised with a series of HFSS simulations. The optimal design parameters were as follows: *W* = 45 mm, *W*_*d*_ = 13.5 mm, *L*_*d*_ = 44 mm, *C* = 5.5 mm, *W*_*c*_ = 0.5 mm, *L*_*c*_ = 6 mm, *G*_*c*_ = 0.5 mm, *W*_*f*_ = 1.0 mm, *D*_*f*_ = 2.0 mm, *G*_*1*_ = 0.4 mm, *G*_*2*_ = 0.5 mm, and *h*_*s*_ = 0.508 mm. The proposed antenna design procedure is described as follows:

1. Two fat dipole arms with lengths equal to the first resonant wavelength of the desired frequency are designed orthogonally to each other. A dipole with a wide arm width generates a second resonant mode. A stripline is bent to connect the two perpendicular dipole arms for CP generation. The crossed-dipole arm is duplicated, and the duplicate dipole arm is rotated to 180° on the same plane as the original on a Rogers RO4003 dielectric substrate.
2. The corners of each fat dipole arm are cut into a triangular shape to generate two orthogonal current paths with a different resonant frequency, which generates another AR band that significantly widens the AR bandwidth.
3. To reduce the effect of the leakage current from the unbalanced transmission line, the power is supplied to the crossed dipole antenna using a microstrip-to-parallel stripline balun. The antenna and the balun are connected with metal pins.
4. Finally, the design parameters of the antenna are tuned or adjusted to achieve their optimal values for improving the impedance and AR bandwidths.

The uniplanar crossed-dipole antenna shows wideband performance characteristics in terms of impedance and AR bandwidths. The impedance bandwidth is 6.55 GHz (2.53–9.08 GHz), which is equivalent to a fractional bandwidth of 112.8%. The antenna produced three AR bands corresponding to the AR minimums at 3.0 GHz, 5.0 GHz, and 7.0 GHz. The AR bands were adjacent to each other and were merged to produce a wide AR bandwidth of 4.83 GHz (2.68–7.51 GHz), which is equivalent to a fractional bandwidth of 94.8%.

### Operation mechanism

#### Fat-crossed dipole antenna

**Figure 2:**
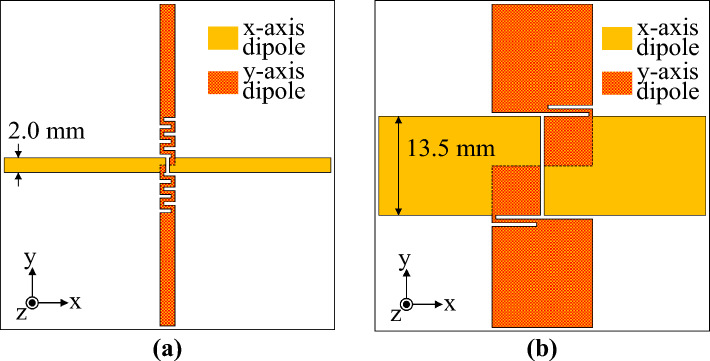
Geometry of the two crossed dipoles: (**a**) narrow arm crossed dipole and (**b**) fat crossed dipole.

**Figure 3:**
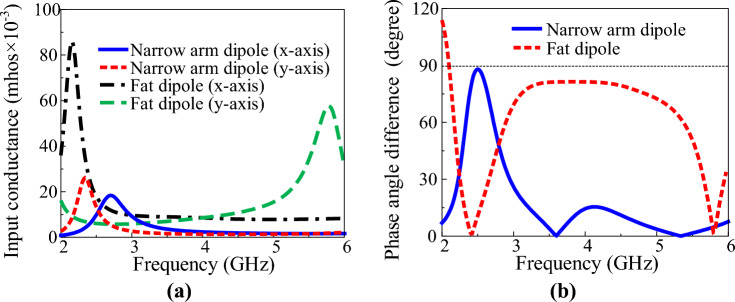
Input admittance characteristics of the dipoles: (**a**) input conductance and (**b**) phase-angle difference between the x- and y-axis dipoles.

**Figure 4:**
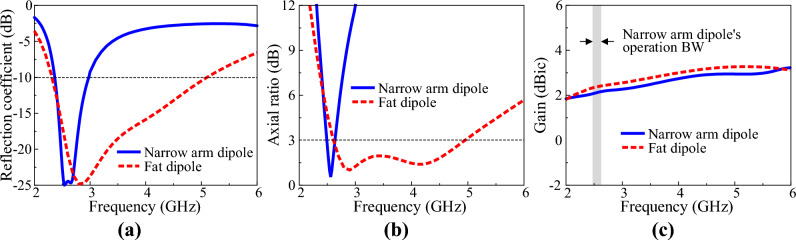
Characteristics of the two crossed dipoles: (**a**) reflection coefficient, (**b**) AR and (**c**) gain.

A crossed-dipole antenna has a structure in which two orthogonal dipoles are connected in parallel. The crossed-dipole antenna generates circular polarisation when the real part of the input admittance of each dipole constituting the antenna is similar and the phase-angle difference of the input admittance of each dipole is close to 90°^6^. Therefore, the change in input impedance of the two orthogonal dipoles should be minimal even if the frequency changes; moreover, the phase-angle difference should be constant for the crossed-dipole antenna to have wide impedance and AR bandwidths. A crossed dipole with a wide bandwidth was implemented using a fat dipole^30^ with little change in input impedance according to frequency change. Figure 2 shows a crossed-dipole antenna with an antenna width *W*_*d*_ = 2 mm and *W*_*d*_ = 13.5 mm. The two orthogonal dipoles were connected by a stripline. In the design of the crossed dipole with *W*_*d*_ = 2 mm, a meander stripline, which is advantageous for impedance matching due to the multiple design parameters, was used. The overall length of the meander stripline was adjusted through the simulation so the antenna could generate CP. The fat crossed dipole has a wide dipole arm width, so the two dipoles are connected using an embedded bent stripline instead of a meander stripline. Impedance and AR bandwidths were optimized by adjusting the length of the bent stripline. To clearly show the reason the fat crossed dipole has a wide AR bandwidth, the crossed-dipole antenna was divided into two parts, as shown in Fig. 2, and the input admittance of the x- and y-axis dipoles was confirmed through the simulation. Figure 3a shows the input conductance of each dipole of the two crossed dipoles. The input conductance of the dipole has a significant impact on the impedance and AR bandwidths of the antenna. First, for impedance matching, the input impedance of the crossed-dipole antenna must be close to 50 Ω. Therefore, for the reflection coefficient of the antenna to be − 10 dB or less, the sum of the input conductance of the x- and y-axis dipoles of the crossed dipole must be close to 2 × 10^−2^ mhos. Next, for the AR of the crossed-dipole antenna to be within 3 dB, the ratio of the two orthogonal electric fields must be within 1:2. In other words, the ratio of power radiated from two orthogonal dipoles must be within 1:4. Therefore, the ratio of the input conductance of the two dipoles must be within 1:4. A crossed-dipole antenna composed of narrow-arm dipoles satisfies the corresponding conditions only around 2.5 GHz. However, a crossed-dipole antenna composed of fat dipoles satisfies the corresponding conditions between about 2.5–5 GHz. The conductance of each dipole of a narrow-arm crossed dipole is similar only in a very narrow frequency band near 2.5 GHz. On the other hand, the two orthogonal dipoles in a fat-crossed dipole have similar conductance in a wide frequency band. Figure 3b shows the phase-angle difference between each dipole of the two crossed dipoles. The input admittance change according to the frequency change in the narrow-arm crossed dipole is immense; therefore, a phase angle difference close to 90° occurs only in a narrow frequency range. On the other hand, the fat dipole generates a phase-angle difference close to 90° in a much wider band because the input admittance change is minimal. Figure 4 shows the characteristics of the two crossed-dipole antennas with different widths. Figure 4a shows the reflection coefficients of the two antennas. The − 10-dB impedance bandwidth of the narrow-arm crossed dipole is 23.9%, whereas the impedance bandwidth of the fat crossed dipole is 75.3%. Figure 4b shows the AR of the antenna. The 3-dB AR bandwidth of the narrow-arm crossed dipole and the fat-crossed dipole is 5.85% and 61.9%, respectively, indicating that the fat-crossed dipole has a much wider AR bandwidth. The gain of the fat-crossed dipole is almost the same as the gain of the narrow-arm crossed dipole. Figure 4c shows the gain of a narrow-arm dipole and a fat dipole. Because the conductor area of a fat dipole is large, the gain is about 0.2 dB higher than that of a narrow-arm dipole at the same frequency.

#### Corner cut fat crossed-dipole antenna

Previously, it has been shown that the impedance and AR bandwidths of a crossed-dipole antenna can be significantly improved by using a fat dipole. To further improve the bandwidth of the antenna, two orthogonal resonance modes were implemented by adding a corner cut to each dipole arm of the fat crossed-dipole antenna, which greatly widened the impedance and AR bandwidths.

**Figure 5:**
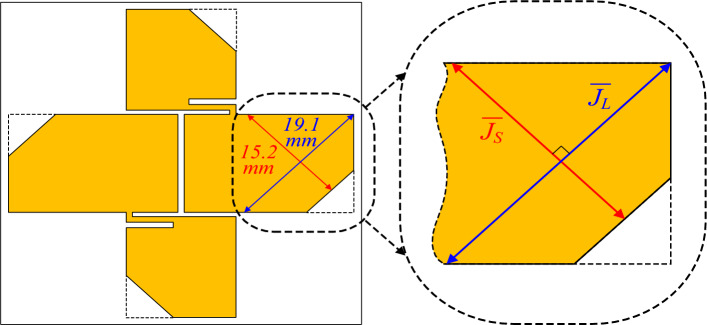
Two orthogonal current paths of the corner-cut fat crossed dipole.

**Figure 6:**
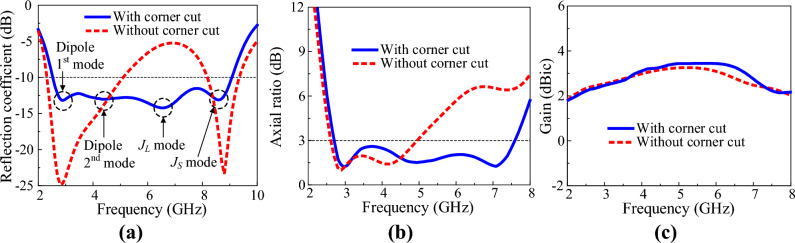
Characteristics of the antennas with and without corner cuts: (**a**) reflection coefficient, (**b**) AR and (**c**) gain.

Figure 5 shows a corner-cut fat crossed-dipole antenna. With the corner cut, two orthogonal current paths with different resonant frequencies can be implemented in the dipole, generating a new AR band at a higher frequency. Figure 6 shows the characteristics of a fat crossed-dipole antenna with and without a corner cut. Figure 6a illustrates the reflection coefficients of the two crossed dipoles. The − 10-dB impedance bandwidths of the antenna with and without corner cuts are 112.8% and 75.3%, respectively. The antenna with a corner cut exhibited two additional resonance modes at a high frequency.

**Figure 7:**
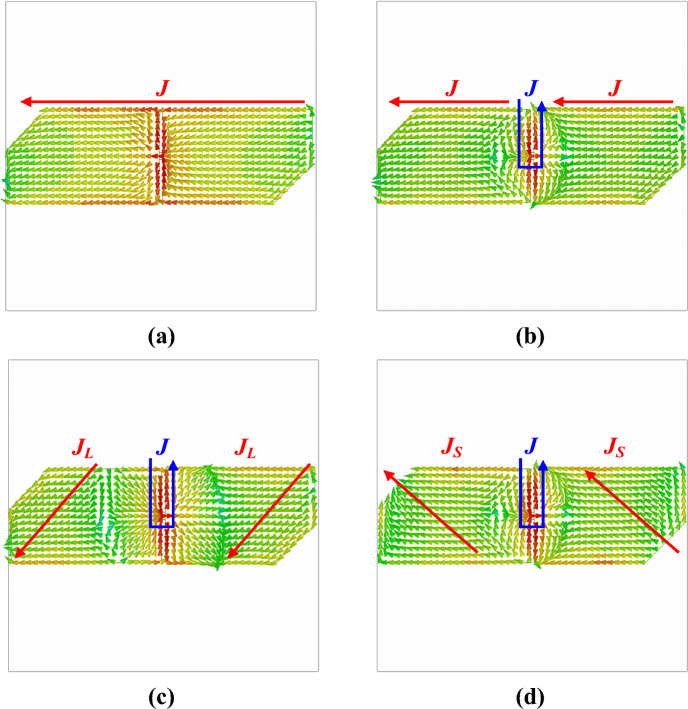
Surface current distribution of corner cut dipole at each resonance frequency: (**a**) 2.5 GHz, (**b**) 4.2 GHz, (**c**) 6.7 GHz, and (**d**) 8.4 GHz.

The physical lengths of the long and short current paths in Fig. 5 are 19.1 and 15.2 mm, respectively. Considering the effective permittivity of the antenna, the resonant frequencies of the long and short current paths are about 6.7 and 8.4 GHz, respectively. Therefore, the two resonant modes at high frequencies of the proposed antenna are generated by the corner cuts. Figure 6b shows the AR of the two crossed-dipole antennas. The 3-dB AR bandwidths of the antenna with and without corner cuts are 94.8% and 61.9%, respectively. The AR minimum point implemented by the orthogonal current paths is formed in the middle of the resonant frequencies of the two current paths. In the corner-cut, crossed-dipole antenna, the third AR minimum point is formed near 7.0 GHz, which is the middle of the resonant frequencies of the two current paths attributed to the corner cut. This greatly widens the AR bandwidth. Figure 6c shows the antenna gain with and without a corner cut. For the antenna without a corner cut, gain reduction begins at 5.5 GHz due to a higher-order mode. However, the antenna with a corner cut begins to reduce the gain at a higher frequency compared to the antenna without a corner cut. The peak gains within the operating band of the two antennas are 3.3 and 3.7 dBic, respectively. To show the effect of the corner cut more clearly, the surface current distribution at each resonant frequency of the corner cut fat dipole is shown in Fig. 7. The red line in the figure is the equivalent current that contributes to radiation, and the blue line is the current that cancels each other out and does not contribute to radiation. Figure 7a,b show that the equivalent current direction is parallel to the dipole axis at the first and second resonant frequencies. Meanwhile, Fig. 7c shows that the equivalent current direction at the third resonant frequency matches the long current path J_L_ implemented by the corner cut. Figure 7d indicates that the equivalent current direction at the fourth resonant frequency matches the short current path J_S_ implemented by the corner cut. Therefore, it is evident that the first and second resonance modes of the antenna are caused by the fat dipole, while the third and fourth resonance modes are caused by the corner cut. The characteristics of the narrow-arm crossed dipole, fat-crossed dipole, and corner-cut fat-crossed dipole are summarised in Table 1.

**Table 1:** Characteristics of three crossed-dipole antennas.

Dipole type	− 10 dB IBW (GHz)	3-dB ARBW (GHz)	Peak gain^1^ (dBic)	Peak gain frequency (GHz)
Narrow-width dipole	2.36–3.00 (23.9%)	2.49–2.64 (5.85%)	2.1	2.64
Fat dipole	2.31–5.10 (75.3%)	2.61–4.95 (61.9%)	3.3	4.95
Corner cut fat dipole	2.53–9.08 (112.8%)	2.68–7.51 (94.8%)	3.7	6.08

*IBW* impedance bandwidth.

^1^Peak gain within the operating band.

#### Surface current distributions and radiation patterns

**Figure 8:**
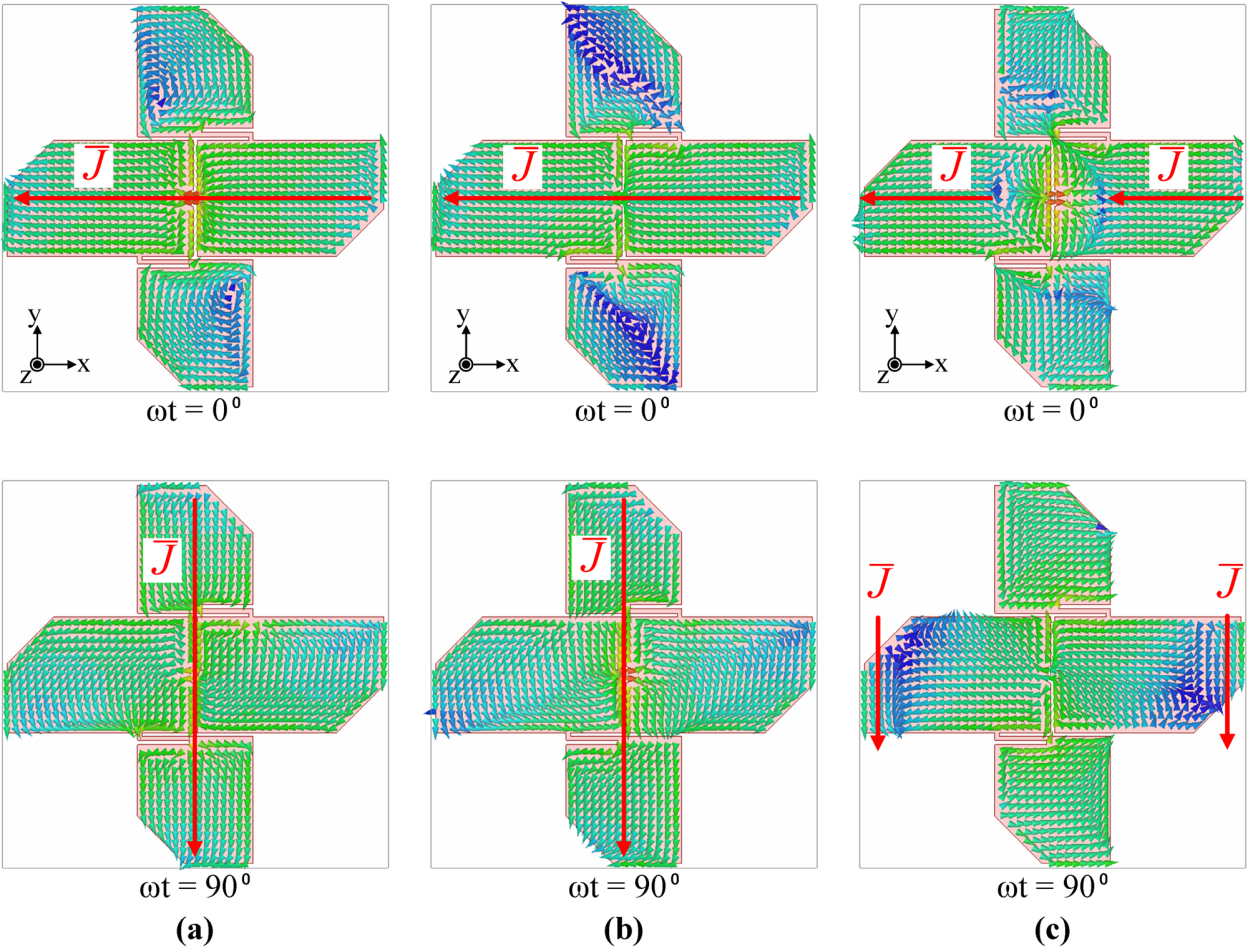
Surface current distributions of the corner-cut fat crossed dipole antenna: (**a**) 3.0 GHz, (**b**) 5.0 GHz, and (**c**) 7.0 GHz.

To better understand the operation mechanism of the antenna, the surface current distributions and radiation patterns of the corner-cut fat crossed-dipole antenna were plotted. Figure 8 shows the antenna surface current distributions at 3.0, 5.0, and 7.0 GHz. Figure 8a demonstrates the surface current distribution of the antenna at 3.0 GHz. When ωt = 0°, a current in the –x direction flows in the x-axis dipole, while a current in the –y direction flows in the y-axis dipole when ωt = 90°; the antenna operates as an RHCP crossed dipole. Figure 8b shows the antenna surface current distribution at 5.0 GHz, and the antenna operates as an RHCP crossed dipole similar to that at 3.0 GHz. Figure 8c shows the antenna surface current distribution at 7.0 GHz, which is an additional CP mode implemented by the corner cut. When ωt = 0°, a current in the –x direction flows through each arm of the x-axis dipole. When ωt = 90°, a current in the –y direction flows at each edge of the x-axis dipole. At 7.0 GHz, the y-axis dipole does not generate circular polarisation, and the two RHCP antennas operate in an arrangement along the x-axis.

The antenna’s current distribution is similar at 3.0 and 5.0 GHz frequencies, so they have almost the same radiation pattern. Meanwhile, the main radiation source of the antenna at 7.0 GHz is an x-axis dipole, which results in a narrowing of the beamwidth in the xz-plane. The proposed antenna has no beam tilting within the bandwidth and has a symmetrical radiation pattern.

## Measurement results

A wideband microstrip-to-parallel strip tapered balun was designed for the antenna measurement. When a coaxial cable, which is an unbalanced transmission line, is used to feed the dipole antenna, the characteristics of the antenna change due to the leakage current^31,32^. It is possible to reduce the leakage current of the coaxial cable by using a sleeve balun. However, because the sleeve balun operates only in a narrow frequency range, it is unsuitable for feeding a broadband antenna. The tapered balun has a simple structure and can reduce the effects of leakage current over a wide frequency range. So it is widely used to feed wideband antennas^33^.

**Figure 9:**
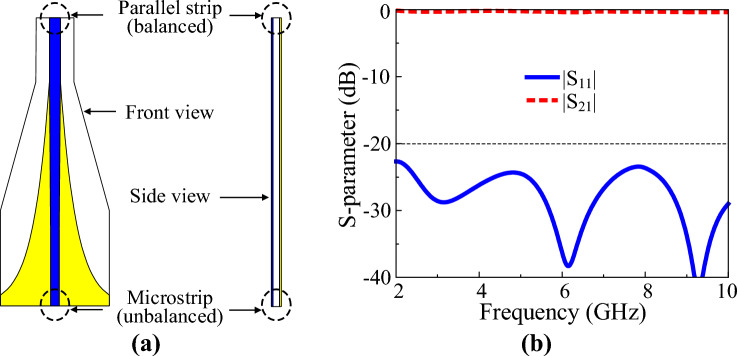
Microstrip-to-parallel strip tapered balun: (**a**) geometry and (**b**) S-parameter characteristics.

**Figure 10:**
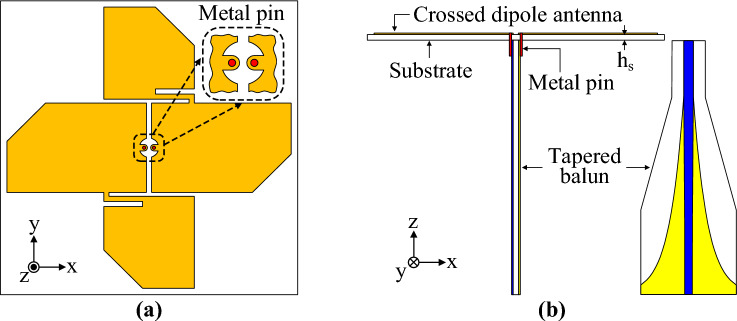
Geometry of the antenna with tapered balun: (**a**) top view and (**b**) side view.

**Figure 11:**
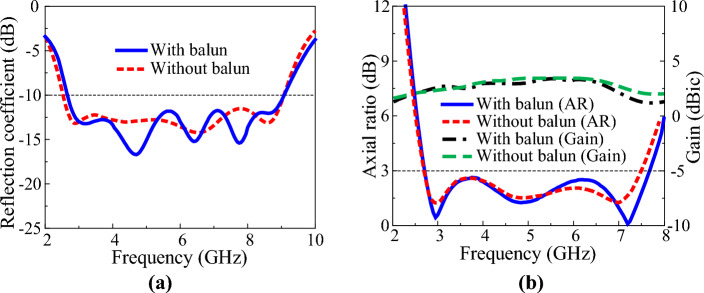
Characteristics of the antenna with and without tapered balun: (**a**) reflection coefficient, (**b**) axial ratio and gain.

Figure 9 shows the geometry and characteristics of the designed tapered balun. The tapered balun has excellent characteristics, with a reflection coefficient of less than –20 dB in the range 2–10 GHz. Figure 10 shows the antenna structure with the proposed crossed dipole and tapered balun. The crossed dipole and the tapered balun are connected by metal pins. To eliminate parasitic capacitance occurring at the connection point between the tapered balun and the antenna feed position, the area around the connection point was cut into a small circular shape. Other design parameters of the antenna remain the same. Figure 11 shows the simulation results of the antenna with the tapered balun and the antenna without the tapered balun. The –10 dB impedance bandwidth of the antenna with a balun is 2.57–9.08 GHz, which is almost equivalent to the impedance bandwidth of the antenna without a balun, 2.53–9.08 GHz. The 3-dB AR bandwidth of the antenna with a balun was 2.69–7.73 GHz, which was slightly wider than the bandwidth of the antenna without a balun, 2.68–7.51 GHz. The peak gains of the antenna with and without balun are 3.5 dBic and 3.6 dBic, respectively, which shows a slight decrease in gain due to the small power loss to the balun.

**Figure 12:**
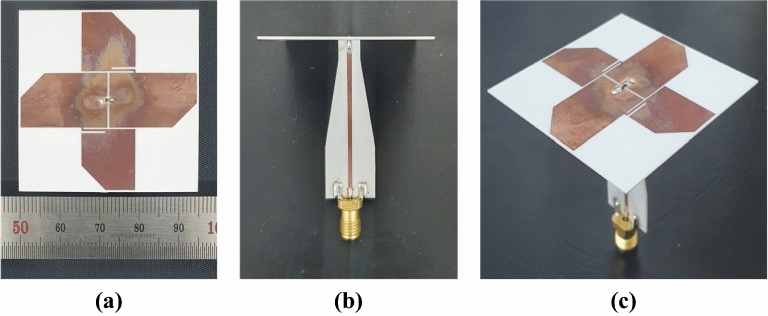
Image of the fabricated sample of uniplanar crossed dipole antenna: (**a**) top view, (**b**) front view, and (**c**) 3-D view.

**Figure 13:**
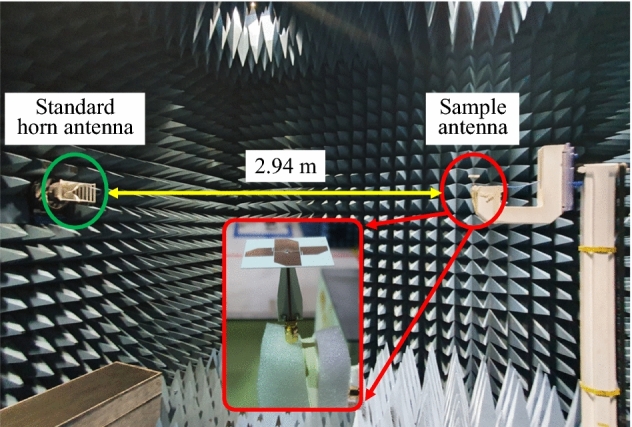
Experimental setup.

**Figure 14:**
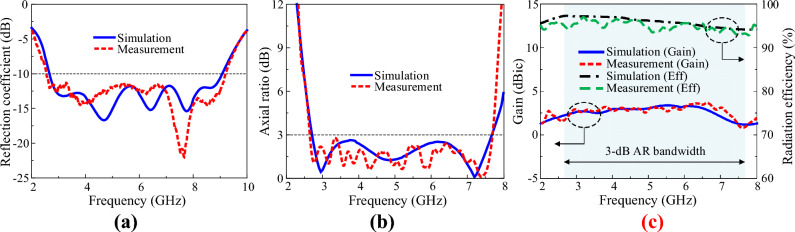
Simulated and measured results of the crossed-dipole antenna: (**a**) reflection coefficient, (**b**) axial ratio, (**c**) gain and radiation efficiency.

The optimised antenna with a balun was fabricated and measured for verification. Figure 12 shows a fabricated sample of the crossed-dipole antenna printed on the same plane of a thin RO4003 dielectric substrate sheet via standard wet-etching technology. Figure 13 shows the experimental setup. The sample antenna was measured in a full anechoic chamber with 5.5 m (W) × 5.5 m (L) × 5.0 m (H) dimensions. The distance between the sample antenna and the standard horn antenna was 2.94 m. As shown in Fig. 14a, the measured and simulated |S_11_| were in good agreement; the measured impedance bandwidth for |S_11_|< − 10 dB was 2.53–9.14 GHz (113.3%), and the simulated bandwidth was 2.57–9.08 GHz (111.8%). The AR was measured at several frequency points, and the results are shown in Fig. 14b. The figure shows that the simulated and measured AR are very similar. However, small fluctuations are observed, which result from fabrication imperfections, the foams, racks, and tape used in the measurement setup, as well as various influences during the measurement process. The measured AR bandwidth spanned from 2.65 to 7.75 GHz (98.1%) and the simulated from 2.69 to 7.73 GHz (96.7%). The simulated and measured broadside gain are shown in Fig. 14c. The measured gain was satisfactory and showed a profile similar to that of the simulated. The measured gain had a peak of 3.7 dBic, and within the 3-dB AR bandwidth, the gain was in the range 0.8–3.7 dBic. The measured radiation efficiency of the antenna is over 93% within the operating bandwidth.

**Figure 15:**
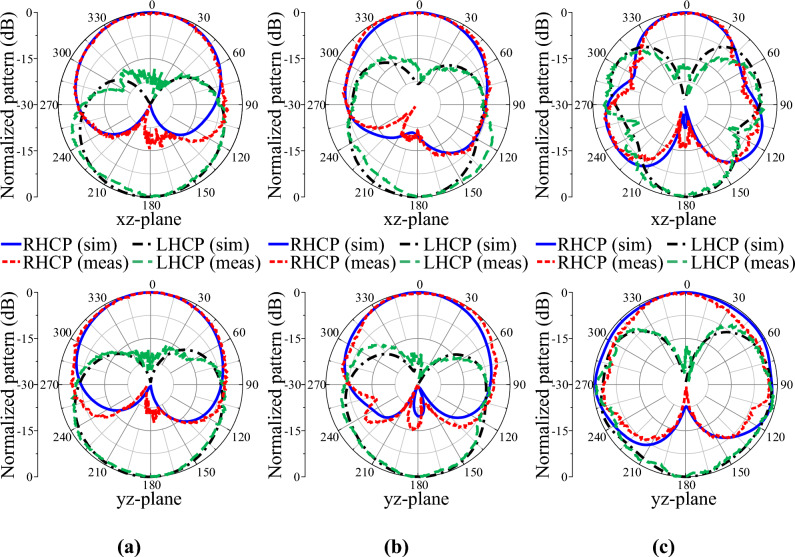
Simulated and measured radiation patterns of the crossed-dipole antenna: (**a**) 3.0 GHz, (**b**) 5.0 GHz, and (**c**) 7.0 GHz.

The radiation patterns were measured at the three frequencies of 3.0 GHz, 5.0 GHz, and 7.0 GHz, as shown in Fig. 15. Good broadside bidirectional CP radiation patterns were produced by the fabricated antenna. The measured patterns in both the x–z and y–z planes were almost symmetric and very similar to the simulated counterparts. The measured patterns demonstrated low cross-polarization levels. The proposed antenna recorded half-power beamwidths (HPBWs) of 102° and 120° at 3.0 GHz, 103° and 107° at 5.0 GHz, and 54° and 99° at 7.0 GHz in the x–z and y–z planes, respectively.

### Comparison

The characteristics of the proposed antenna and other CP antennas were compared. The antennas in Refs.^1,9,23^ are sequentially rotated feed antennas using four dipoles as radiation elements. Sequentially rotated feed dipole antennas have a high gain due to a large number of dipoles. However, the bandwidth of the radiation element itself is narrow, which limits the AR bandwidth. In addition, the radiation efficiency of the antenna is reduced because a feeding structure is required to implement the circular polarization. The structures in Refs.^2,10^ are conventional biplanar crossed-dipole antennas using two orthogonal dipoles. A conventional crossed-dipole antenna is easy to miniaturise and has high radiation efficiency, but has a low gain; moreover, the input impedance of each dipole varies greatly, so the bandwidth is very narrow. The structure given in Ref.^34^ is a broadband CP monopole antenna. A CP monopole antenna has very wide impedance and AR bandwidths; however, a 3-dB gain bandwidth is narrower than an AR bandwidth, and the radiation pattern change according to frequency change is severe. In addition, the antenna structure is very complex, making it difficult to design and optimise. The proposed antenna has a simpler structure than existing antennas, can realise a wide bandwidth, and has a stable gain within the AR bandwidth. In addition, the radiation efficiency is higher than 93% within the AR bandwidth and the radiation pattern within the bandwidth is symmetrical. The characteristics of the conventional CP antennas and the proposed crossed-dipole antenna are summarised in Table 2.

**Table 2:** Performance comparison of the proposed antenna with other antenna designs.

Antenna structure	Antenna type	Size (λ_L_^3^)	IBW (%)	AR BW (%)	Peak gain (dBic)	Rad. Eff (%)
Ref.^1^^1^	dipole	0.41 × 0.41 × 0.0040	31.9	21.56	5.0	> 90
Ref.^2^^1^	dipole	0.35 × 0.35 × 0.0052	16.4	11.7	1.9	> 93
Ref.^9^^2^	dipole	0.43 × 0.43 × 0.0042	5.6	5.6	4.5	> 85
Ref.^10^^1^	dipole	0.18 × 0.18 × 0.0015	10.04	2.08	1.4	N/A
Ref.^23^^1^	dipole	0.59 × 0.59 × 0.02	1.27	1.27	6	N/A
Ref.^34^^1^	monopole	0.33 × 0.56 × 0.01	106.3	104.7	6.4	N/A
Proposed design^1^	dipole	0.4 × 0.4 × 0.0045	113.3	98.1	3.7	> 93

*λ*_*L*_ lowest frequency in 3-dB AR bandwidth, *IBW* impedance bandwidth.

^1^|S_11_|≤ − 10 dB.

^2^|S_11_|≤ − 15 dB.

## Conclusion

In this study, a uniplanar crossed-dipole antenna comprising a corner-cut fat dipole and a bent stripline is presented. The proposed antenna is different from other crossed-dipole antennas in that both crossed-dipole arms are printed on the same plane of the substrate. The bent striplines connecting the dipole arms are designed for CP generation, and the corner-cut fat dipoles are significant in impedance matching and AR bandwidth broadening. The antenna produced multiple resonances in its reflection coefficient profile and three AR bands that combine to give the antenna its broadband characteristics. The broadband properties of the antenna were numerically and experimentally verified to validate the antenna design. The simulated and measured performances of the proposed antenna were in good agreement. The small size of the antenna, planar configuration, and broadband CP characteristics make the antenna a suitable candidate for many wireless communication systems.

## Data Availability

The datasets generated during and/or analyzed during the current study are available from the corresponding author on reasonable request.
